# Bacterial infections in patients with COVID-19: the impact of procalcitonin testing on antibiotics prescription in the real world

**DOI:** 10.1186/s12879-023-08849-x

**Published:** 2024-01-19

**Authors:** Grace CY Lui, Catherine SK Cheung, Terry CF Yip, Mandy SM Lai, Timothy CM Li, Grace LH Wong

**Affiliations:** 1 Department of Medicine and Therapeutics, Faculty of Medicine, The Chinese University of Hong Kong, 9/F Lui Che Woo Clinical Sciences Building, Hong Kong SAR, China; 2 Medical Data Analytics Centre (MDAC), The Chinese University of Hong Kong, Hong Kong SAR, China; 3 Institute of Digestive Disease, The Chinese University of Hong Kong, Hong Kong SAR, China

**Keywords:** Procalcitonin, Antibiotics stewardship, COVID-19, Bacterial Infections

## Abstract

**Background:**

Bacterial infections are not prevalent among patients hospitalized with COVID-19, while unnecessary prescription of antibiotics was commonly observed. This study aimed to determine the impact of procalcitonin testing on antibiotics prescription in the real-world setting.

**Methods:**

We performed a territory-wide retrospective cohort study involving all laboratory-confirmed patients hospitalized in public hospitals in Hong Kong in 2020 with COVID-19. We determined the prevalence of bacterial co-infections (documented infections within 72 h of admission) and secondary bacterial infections (infections after 72 h of admission) and antibiotics consumption, and the correlation between procalcitonin testing and antibiotics prescription.

**Results:**

The cohort included 8666 patients, with mean age 45.3 ± 19.9 years, 48.5% male, and comorbidities in 26.9%. Among 2688 patients with bacterial cultures performed, 147 (5.5%) had bacterial co-infections, and 222 (8.3%) had secondary bacterial infections. Antibiotics were prescribed for 2773 (32.0%) patients during the hospital admission. Procalcitonin tests were performed for 2543 (29.3%) patients. More patients with procalcitonin testing received antibiotics (65.9% vs. 17.9%, p < 0.001). Procalcitonin testing was associated with 5-fold increased risk of antibiotics prescription after adjusting for confounding variables. At hospital level, procalcitonin testing correlated with antibiotics prescription. Patients with procalcitonin level < 0.5 ng/mL had a lower probability of antibiotics initiation and shorter duration of antibiotics therapy.

**Conclusions:**

Procalcitonin testing was not associated with lower prescription of antibiotics. Patients with low procalcitonin level had lower antibiotics exposure, supporting the use of procalcitonin to exclude bacterial infections aiding early stopping of antibiotics among patients hospitalized with COVID-19.

**Supplementary Information:**

The online version contains supplementary material available at 10.1186/s12879-023-08849-x.

## Background

Bacterial infections are not uncommon among patients hospitalized with respiratory viral infections. Up to 30% of patients hospitalized for influenza infections had bacterial co-infections during the course of hospital admission [1–3]. The presence of bacterial infections was associated with higher mortality and poorer outcomes among patients with respiratory viral infections [2, 3]. On the other hand, evidence has shown that concomitant bacterial infections were more than 3-fold less prevalent among patients hospitalized for COVID-19 than in those with influenza [4].

Several meta-analyses and systematic reviews have shown that the overall pooled prevalence of bacterial infections among patients hospitalized with COVID-19 ranged from 4 to 9% [5–9]. Bacterial co-infections, which were detected at the time of hospital admission, occurred in 3–5% [5–7]; while secondary, or hospital-acquired bacterial infections, occurred in 4–22%, with a pooled prevalence of 13–14% [5–7]. The most common bacterial infections were respiratory tract infections, followed by bloodstream and urinary tract infections [8, 10]. Bacterial infections were more common among critically ill patients treated in intensive care units or on mechanical ventilation [5–7, 9], and in patients with advanced age and comorbidities [7, 11]. As in other respiratory viral infections, the presence of bacterial infections was associated with longer length of stay in hospital and higher mortality [4, 7, 12].

Despite the low prevalence of bacterial infections among patients hospitalized with COVID-19 disease, a high proportion of patients had received antibiotics. Meta-analyses and systematic reviews showed that 60–98% of hospitalized patients had received antibiotics [5–9]. Prescription of unnecessary broad-spectrum antibiotics was common and consistent over time [5, 6, 10], contributing significantly to the accelerating threat of antimicrobial resistance globally [13].

The low prevalence of confirmed bacterial infections among patients hospitalized with COVID-19 suggested that most of the prescription of antibiotics was likely to be inappropriate. Antibiotic stewardship strategies to optimize antibiotics use among patients hospitalized with COVID-19 are thus strongly indicated [13]. The most optimal strategy should be feasible to be implemented even when the burden on hospital manpower and resources is immense during COVID-19 waves.

The use of procalcitonin as a blood test is an attractive option as a tool for antibiotic stewardship in such scenarios. Procalcitonin is a precursor peptide of calcitonin and a cytokine mediator, is elevated in systemic bacterial infections, and shows higher diagnostic accuracy compared with other biomarkers for severe infections [14]. As a tool for antibiotic stewardship, procalcitonin has been shown to facilitate the reduction in antibiotics exposure for patients with acute respiratory infections, by both reducing initiation of antibiotics and shortening the duration of antibiotics therapy [15].

Therefore, we performed this study to determine the prevalence, risk factors and outcomes of bacterial co-infections and secondary infections among hospitalized patients with COVID-19 in Hong Kong. We also aimed to determine the impact of procalcitonin testing on antibiotic consumption among patients hospitalized with COVID-19.

## Methods

### Study design and study population

We performed a territory-wide retrospective cohort study involving all patients hospitalized for COVID-19 in public hospitals in Hong Kong in 2020. We aimed to determine the prevalence of bacterial infections and the impact of procalcitonin testing on antibiotics prescription. We included all patients with laboratory-confirmed SARS-CoV-2 infection hospitalized in all public hospitals in Hong Kong from January 2020 to December 2020. All patients were followed for 90 days from the first day of hospital admission. The study was approved by Joint Chinese University of Hong Kong – New Territories East Cluster (NTEC) Cluster Research Ethics Committee (2020.467).

### Study procedures

We collected demographic and clinical data from the Hospital Authority’s Clinical Data Analysis and Reporting System (CDARS). We recorded data on age, sex, comorbidities, intensive care unit admission, baseline laboratory parameters, including procalcitonin, white cell count, neutrophil count, C reactive protein, lactate dehydrogenase, bilirubin, alanine transaminase, and creatinine, and antibiotics consumption. We also documented length of stay in hospital, all-cause mortality and re-admission to hospital within 90 days after hospital discharge.

Procalcitonin testing has been introduced to public hospitals in Hong Kong since October 2018. Different hospitals have varied practice in the access right of requesting procalcitonin testing, and whether the results were followed by Antibiotic Stewardship teams in guiding initiation and cessation of antibiotics. We recorded all procalcitonin tests performed for each patient and the dates of tests and results of procalcitonin. We also recorded the starting and ending dates and doses of all antibiotics prescribed for all patients during the hospital admission.

### Endpoints

The primary endpoint was the prevalence of bacterial infections among hospitalized patients with bacterial cultures performed in one or more specimens. Bacterial infections included (i) bacterial co-infections, defined as documented bacterial infections within 72 h of hospital admission, and (ii) secondary bacterial infections, defined as documented bacterial infections after 72 h of hospital admission up to 90 days after admission. Only patients with one or more bacterial cultures performed were included in the analysis of the primary endpoint.

Secondary endpoints included antibiotic consumption during hospital admission. This was measured by (i) days of therapy, defined as the total number of antibiotic-days of therapy, by calculating the sum of the number of days each patient received each individual antibiotics, (ii) length of therapy, defined as the number of days that each patient had received antibiotics, irrespective of the number of different antibiotics, and (iii) World Health Organization defined daily doses (DDDs).

### Statistical analysis

We presented data as mean ± standard deviation (SD), or median (interquartile range, IQR) according to data distribution. We compared categorical variables between groups using chi-square test, and continuous variables using Student-t test or Mann-Whitney U test, as appropriate.

We determined the association between procalcitonin testing and use of antibiotics using multivariate logistic regression model, adjusting for age, sex and variables associated with use of antibiotics on univariate analyses. We then determined the association between procalcitonin use and antibiotic prescription by hospital level using Spearman correlation.

## Results

### The cohort

A total of 8666 patients hospitalized with laboratory-confirmed COVID-19 during the period January 2020 to December 2020 in seventeen public hospitals in Hong Kong were identified. The mean age was 45.3 ± 19.9 years, 4201 (48.5%) were male, and 2331 (26.9%) had one or more comorbidities. 409 (4.7%) of them required intensive care unit admission, and 159 (1.8%) died during the hospital admission. Table 1 shows the baseline characteristics of this cohort.

**Table 1:** Baseline characteristics of the whole cohort and patients with and without bacterial infections

Characteristics	All N = 8666	Bacterial cultures performed N = 2688	Bacterial infection N = 369	No infection N = 2319	p
Age (years)	45.3 ± 19.9	51.4 ± 19.5	62.4 ± 17.8	49.7 ± 19.1	< 0.001
Male	4201 (48.5%)	1394 (51.9%)	198 (53.7%)	1196 (51.6%)	0.457
Any comorbidities	2331 (26.9%)	1008 (37.5%)	223 (60.4%)	785 (33.9%)	< 0.001
Hypertension	1216 (14.0%)	547 (20.3%)	129 (35.0%)	418 (18.0%)	< 0.001
Diabetes	708 (8.2%)	334 (12.4%)	78 (21.1%)	256 (11.0%)	< 0.001
Obesity	247 (2.9%)	95 (3.5%)	27 (7.3%)	68 (2.9%)	< 0.001
Cardiovascular diseases	356 (4.1%)	178 (6.6%)	51 (13.8%)	127 (5.5%)	< 0.001
Neurological diseases	270 (3.1%)	135 (5.0%)	46 (12.5%)	89 (3.8%)	< 0.001
Liver diseases	252 (2.9%)	103 (3.8%)	18 (4.9%)	85 (3.7%)	0.260
Psychiatric disorders	226 (2.6%)	99 (3.7%)	23 (6.2%)	76 (3.3%)	0.005
Haematological disorders	224 (2.6%)	90 (3.3%)	31 (8.4%)	59 (2.5%)	< 0.001
Haematological and solid organ malignancy	143 (1.7%)	61 (2.3%)	23 (6.2%)	38 (1.6%)	< 0.001
Endocrine disorders	122 (1.4%)	48 (1.8%)	8 (2.2%)	40 (1.7%)	0.550
Pulmonary diseases	118 (1.4%)	49 (1.8%)	14 (3.8%)	35 (1.5%)	0.002
Rheumatological diseases	117 (1.4%)	46 (1.7%)	14 (3.8%)	32 (1.4%)	0.001
Renal diseases	109 (1.3%)	59 (2.2%)	24 (6.5%)	35 (1.5%)	< 0.001
Pregnancy	34 (0.4%)	9 (0.3%)	1 (0.3%)	8 (0.3%)	1.000
Immunocompromised conditions	10 (0.1%)	5 (0.2%)	2 (0.5%)	3 (0.1%)	0.142
Charlson comorbidity index	0 (0, 2)	1 (0, 2)	3 (1, 4)	1 (0, 2)	< 0.001
White cell count (x 10^9^/L)	5.4 (4.3, 6.8)	5.3 (4.2, 6.7)	5.5 (4.3, 7.5)	5.3 (4.2, 6.6)	0.002
Neutrophil count (x 10^9^/L)	3.3 (2.4, 4.4)	3.4 (2.5, 4.5)	3.7 (2.8, 5.4)	3.3 (2.5, 4.4)	< 0.001
C reactive protein (mg/L)	0.39 (0.14, 1.36)	0.65 (0.22, 2.40)	1.91 (0.44, 7.60)	0.55 (1.93, 1.90)	< 0.001
Lactate dehydrogenase (U/L)	193 (165, 234)	200 (169, 248)	224 (184, 321)	198 (168, 240)	< 0.001
Bilirubin (µmol/L)	7.5 (5.5, 10.4)	7.2 (5.1, 10.3)	8.0 (5.6, 11.1)	7.0 (5.1, 10.0)	0.003
Alanine transaminase (U/L)	23 (16, 35)	23 (16, 36)	24 (16, 37)	23 (16, 35)	0.475
Creatinine (µmol/L)	69 (58, 83)	72 (60, 87)	77 (63, 96)	72 (60, 86)	< 0.001
Intensive care	409 (4.7%)	242 (9.0%)	115 (31.2%)	127 (5.5%)	< 0.001

### Prevalence of bacterial Infections

In this cohort, 2688 (31.0%) had one or more bacterial cultures done, including 1992 blood samples, 1343 respiratory samples, 1361 urine samples, and 496 miscellaneous samples. Among these patients, 369 (13.7%) patients had bacterial infections. Among these patients, 147 (5.5%) had bacterial co-infections, with 68 (46.3%) respiratory tract infection, 59 (40.1%) urinary tract infection, 12 (8.2%) bloodstream infection, and 8 (5.4%) other infections; while 222 (8.3%) had secondary bacterial infections, with 120 (54.1%) respiratory tract infection, 75 (33.8%) urinary tract infection, 14 (6.3%) bloodstream infection, and 13 (5.9%) other infections. The bacterial pathogens are presented in Supplementary Table 1.

Table 1 shows the baseline characteristics in patients with and without bacterial infections among those with bacterial cultures performed. On multivariate analyses, patients with bacterial infections were older (adjusted odds ratio [aOR] 1.024, 95% confidence interval [CI] 1.016–1.032, p < 0.001), had more neurological diseases (aOR 2.07, 95% CI 1.34–3.18, p = 0.001), hematological diseases (aOR 2.13, 95% CI 1.26–3.59, p = 0.005), and malignancy (aOR 2.50, 95% CI 1.38–4.52, p = 0.002), higher white cell count (aOR 1.07, 95% CI 1.02–1.13, p = 0.005), and C reactive protein (aOR 1.04, 95% CI 1.02–1.07, p = 0.002), and higher proportion required intensive care (aOR 4.94, 95% CI 3.59–6.80, p < 0.001).

Patients with bacterial infections had higher risk of death (11.9% vs. 2.5%, p < 0.001) and longer length of stay in hospital (22 days, interquartile range [IQR] 14–38 vs. 14 days, IQR 10–19, p < 0.001).

### Antibiotics use

Among the whole cohort of 8666 patients, 2773 (32.0%) had antibiotics prescribed during the hospital admission. The median days of antibiotics therapy was 9 days (IQR 6–15), while the length of antibiotics therapy was 8 days (IQR 6–12). The median DDD was 9.87 (IQR 5.33, 16.58). Bacterial cultures were performed in 58.7% and 18.0% of patients with and without antibiotics prescription respectively. Table 2 shows the baseline characteristics in patients with and without prescription of antibiotics. Patients who were prescribed antibiotics were older, had more comorbidities, higher neutrophil count, C reactive protein, lactate dehydrogenase, alanine transaminase and creatinine levels, and higher proportion required intensive care. These patients also had higher risk of bacterial infections (20.3% vs. 3.7%, p < 0.001), longer length of stay in hospital (median 16 days, IQR 12–24 vs. 11 days, IQR 6–14, p < 0.001), and higher risk of death (5.4% vs. 0.2%, p < 0.001).

**Table 2:** Baseline characteristics of all patients with and without antibiotics prescription

Characteristics	Antibiotics N = 2773	No antibiotics N = 5893	P	Adjusted odds ratio (95% CI)	P
Age (years)	56.6 ± 17.8	40.0 ± 18.5	< 0.001	1.022 (1.018, 1.026)	< 0.001
Male	1452 (52.4%)	2749 (46.6%)	< 0.001		
Any comorbidities	1276 (46.0%)	1055 (17.9%)	< 0.001		
Hypertension	730 (26.3%)	486 (8.2%)	< 0.001	1.243 (1.045, 1.478)	0.014
Diabetes	444 (16.0%)	264 (4.5%)	< 0.001		
Obesity	145 (5.2%)	102 (1.7%)	< 0.001		
Cardiovascular diseases	242 (8.7%)	114 (1.9%)	< 0.001	1.429 (1.057, 1.930)	0.020
Neurological diseases	198 (7.1%)	72 (1.2%)	< 0.001	2.730 (1.954, 3.814)	< 0.001
Liver diseases	125 (4.5%)	127 (2.2%)	< 0.001		
Psychiatric disorders	112 (4.0%)	114 (1.9%)	< 0.001	1.547 (1.107, 2.162)	0.011
Haematological disorders	112 (4.0%)	112 (1.9%)	< 0.001		
Haematological and solid organ malignancy	87 (3.1%)	56 (1.0%)	< 0.001		
Endocrine disorders	66 (2.4%)	56 (1.0%)	< 0.001		
Pulmonary diseases	69 (2.5%)	49 (0.8%)	< 0.001	1.943 (1.199, 3.150)	0.007
Rheumatological diseases	67 (2.4%)	50 (0.8%)	< 0.001		
Renal diseases	83 (3.0%)	26 (0.4%)	< 0.001		
Pregnancy	10 (0.4%)	24 (0.4%)	0.746		
Immunocompromised conditions	7 (0.3%)	3 (0.1%)	0.015		
Charlson comorbidity index	2 (0, 3)	0 (0, 1)	< 0.001		
White cell count (x 10^9^/L)	5.4 (4.3, 6.8)	5.4 (4.3, 6.8)	0.881		
Neutrophil count (x 10^9^/L)	3.5 (2.6, 4.7)	3.2 (2.4, 4.3)	< 0.001		
C reactive protein (mg/L)	1.21 (0.38, 4.20)	0.30 (0.10, 0.69)	< 0.001	1.254 (1.211, 1.299)	< 0.001
Lactate dehydrogenase (U/L)	215 (180, 277)	184 (160, 216)	< 0.001	1.002 (1.001, 1.003)	0.004
Bilirubin (µmol/L)	7.4 (5.4, 10.3)	7.6 (5.6, 10.5)	0.163		
Alanine transaminase (U/L)	25 (17, 38)	22 (16, 34)	< 0.001		
Creatinine (µmol/L)	74 (62, 90)	67 (56, 80)	< 0.001	1.004 (1.001, 1.006)	0.004
Intensive care	376 (13.6%)	33 (0.6%)	< 0.001	5.800 (3.672, 9.162)	< 0.001
Procalcitonin use	1676 (60.4%)	867 (14.7%)	< 0.001	5.250 (4.659, 5.916)	< 0.001

### Procalcitonin testing, bacterial Infections, and antibiotics consumption

Among the whole cohort, 2543 (29.3%) patients had one or more procalcitonin tests performed. The median number of procalcitonin tests per patient was 1 (IQR 1–3). Among those with procalcitonin tests performed, 1320 (51.9%) had 1 procalcitonin test, 749 (29.4%) had 2 or 3 tests, and 474 (18.6%) had more than 3 tests. Patients with procalcitonin testing were older, had more comorbidities, higher neutrophil count, higher C reactive protein, and higher prevalence of bacterial infections, and a higher proportion required intensive care (Supplementary Table 2).

Among the 1334 patients with procalcitonin testing and bacterial cultures performed, 251 (18.8%) had bacterial infections. Using 0.5 ng/mL as cut-off, the sensitivity of procalcitonin in detecting bacterial infection was 37.9% (95% CI 31.8% − 44.2%), specificity was 94.1% (95% CI 92.5% − 95.4%), positive predictive value was 59.8% (95% CI 51.7% − 67.4%), and negative predictive value was 86.7% (95% CI 84.7% − 88.6%).

More patients with procalcitonin testing had received antibiotics (65.9% vs. 17.9%, p < 0.001). On multivariate analyses, procalcitonin testing was independently associated with a 5.25-fold higher risk of antibiotics prescription (aOR 5.25, 95% CI 4.66–5.92, p < 0.001), after adjusting for other confounding variables (Table 2). Patients who had procalcitonin testing had longer days of antibiotics therapy (median 11 days, IQR 7–19 vs. median 7 days, IQR 4–10, p < 0.001), longer length of antibiotics therapy (median 9.5 days, IQR 7–15 vs. median 7 days, IQR 4–8, p < 0.001), and higher DDD consumption (median 11.58, IQR 6.67–20.49 vs. median 8.00, IQR 4.00, 11.60, p < 0.001).

Among those with procalcitonin tests, 2312 (90.9%) had procalcitonin level < 0.5 ng/mL in all tests. Among those with procalcitonin tests performed, procalcitonin level < 0.5 ng/mL was independently associated with a lower probability of antibiotics prescription (aOR 0.046, 95% CI 0.006–0.336, p = 0.002), after adjusting for other confounding variables (Table 3). Patients with procalcitonin level < 0.5 ng/mL had shorter days of antibiotics therapy (median 10 days, IQR 7–16 vs. median 30 days, IQR 15–57, p < 0.001), shorter length of antibiotics therapy (median 9 days, IQR 7–13 vs. median 21 days, IQR 11–36, p < 0.001), and lower DDD consumption (media 10.67, IQR 6.00-17.33 vs. median 27.99, IQR 13.48–55.31, p < 0.001).

**Table 3:** Baseline characteristics of patients who had procalcitonin testing with and without antibiotics prescription

Characteristics	Antibiotics N = 1676	No antibiotics N = 867	P	Adjusted odds ratio (95% CI)	P
Age (years)	60.1 ± 16.2	44.2 ± 19.4	< 0.001	1.031 (1.025, 1.037)	< 0.001
Male	931 (55.5%)	386 (44.5%)	< 0.001		
Any comorbidities	860 (51.3%)	225 (26.0%)	< 0.001		
Hypertension	508 (30.3%)	110 (12.7%)	< 0.001		
Diabetes	325 (19.4%)	64 (7.4%)	< 0.001		
Obesity	103 (6.1%)	14 (1.6%)	< 0.001		
Cardiovascular diseases	162 (9.7%)	23 (2.7%)	< 0.001		
Neurological diseases	117 (7.0%)	11 (1.3%)	< 0.001	3.022 (1.524, 5.991)	0.002
Liver diseases	90 (5.4%)	32 (3.7%)	0.060		
Psychiatric disorders	68 (4.1%)	18 (2.1%)	0.009	1.847 (1.016, 3.356)	0.044
Haematological disorders	67 (4.0%)	33 (3.8%)	0.814		
Haematological and solid organ malignancy	62 (3.7%)	16 (1.8%)	0.010		
Endocrine disorders	41 (2.4%)	14 (1.6%)	0.172		
Pulmonary diseases	40 (2.4%)	5 (0.6%)	0.001		
Rheumatological diseases	49 (2.9%)	13 (1.5%)	0.027		
Renal diseases	54 (3.2%)	8 (0.9%)	< 0.001	0.282 (0.106, 0.749)	0.011
Pregnancy	5 (0.3%)	6 (0.7%)	0.201		
Immunocompromised conditions	7 (0.4%)	2 (0.2%)	0.727		
Charlson comorbidity index	2 (1, 3)	0 (0, 2)	< 0.001		
White cell count (x 10^9^/L)	5.5 (4.4, 6.9)	5.2 (4.2, 6.6)	0.004	0.903 (0.822, 0.993)	0.035
Neutrophil count (x 10^9^/L)	3.7 (2.7, 4.9)	3.2 (2.4, 4.2)	< 0.001	1.181 (1.047, 1.331)	0.007
C reactive protein (mg/L)	1.59 (0.46, 5.50)	0.34 (0.11, 0.96)	< 0.001	1.226 (1.152, 1.305)	< 0.001
Lactate dehydrogenase (U/L)	225 (188, 297)	192 (166, 229)	< 0.001		
Bilirubin (µmol/L)	7.6 (5.6, 10.6)	7.0 (5.0, 10.0)	0.001	0.970 (0.949, 0.991)	0.005
Alanine transaminase (U/L)	26 (17, 40)	23 (16, 35)	< 0.001		
Creatinine (µmol/L)	77 (63, 93)	67 (56, 82)	< 0.001	1.011 (1.006, 1.016)	< 0.001
Intensive care	341 (20.3%)	8 (0.9%)	< 0.001	7.225 (3.461, 15.080)	< 0.001
Procalcitonin < 0.5 ng/mL	1447 (86.3%)	865 (99.8%)	< 0.001	0.046 (0.006, 0.336)	0.002

Among the 1676 patients with both antibiotics prescribed and procalcitonin tests performed, 398 (23.7%) had procalcitonin tests performed prior to the initiation of antibiotics. The median time from first procalcitonin result < 0.5 ng/mL to the cessation of antibiotics was 8 (IQR 4, 14) days, and only 191 (11.7%) of patients had antibiotics stopped within 2 days of a low procalcitonin result.

**Fig. 1:**
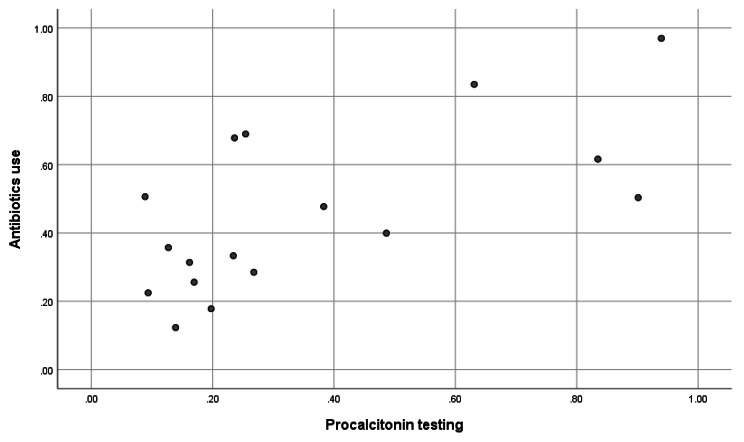
Correlation between procalcitonin testing and antibiotics use by hospital

Regarding the association of antibiotics use and procalcitonin testing by individual hospitals, procalcitonin testing ranged from 8.86 to 93.9%, and procalcitonin testing positively correlated with antibiotics prescription (Spearman’s rho coefficient 0.600, p = 0.011) (Fig. 1).

## Discussion

In this territory-wide retrospective cohort study, 5.5% of patients hospitalized with COVID-19 had bacterial co-infections and 8.3% had secondary bacterial infections, with the most common being respiratory tract infections. 32% had antibiotics prescribed during hospital admission. Procalcitonin testing was associated with higher antibiotics prescription, while a low procalcitonin level was associated with lower probability of antibiotics initiation and shorter duration of antibiotics therapy.

The prevalence of bacterial infections among patients hospitalized with COVID-19 in Hong Kong was low, and was similar to other countries for both co-infections and secondary infections [5–7]. Risk factors included older age and underlying comorbidities, including neurological diseases, hematological diseases and malignancy. As in other studies [4, 7], patients with bacterial infections had higher risk of prolonged hospital stay and mortality.

In our cohort, 32% of patients were prescribed antibiotics during the hospital stay. This was much lower than the pooled prevalence of antibiotics prescription ranging from 60 to 98% among hospitalized patients in recent systematic reviews and meta-analyses [5–9]. A possible reason for the lower probability of antibiotics prescription was the inclusion of judicious antibiotics use in the local clinical management recommendation for COVID-19 in public hospitals. However, this proportion of antibiotics prescription was nonetheless higher than the prevalence of documented bacterial infections in the cohort, suggesting inappropriate initiation of antibiotics treatment in some patients, and room for optimizing antibiotics prescription in patients hospitalized with COVID-19 [6].

Expert opinion has recommended empirical antibiotics only in critically ill patients, patients with severe immunosuppression, radiographic features of bacterial pneumonia, or laboratory parameters of bacterial infection [7]. However, due to overlap of clinical presentation between COVID-19 and bacterial pneumonia, making decisions on initiation and duration of antibiotics based solely on clinical parameters may be challenging for clinicians. Both total white cell count and C reactive protein at baseline were independently associated with documented bacterial infections in our cohort. However, although these biomarkers were often elevated in bacterial infections, they were far from ideal in diagnosing bacterial infections in patients with COVID-19, as they have demonstrated poor ability as biomarkers for detection of bacterial infections [16].

Microbiological diagnostic tools, such as conventional cultures and multiplex molecular tests, are also considered when making decisions on prescribing antibiotics therapy, although reliance on positive cultures may underestimate bacterial coinfections and molecular tests may not be able to differentiate colonization from true infection [11]. In our cohort, more than 40% of patients with antibiotics prescription did not have bacterial cultures performed, demonstrating the challenges in obtaining appropriate specimens for bacterial cultures in real-world settings.

The use of procalcitonin testing in guiding antibiotics prescription has been studied in the setting of COVID-19. Although procalcitonin was significantly elevated in patients with concomitant bacterial infections, it was shown to be a poor predictor of bacterial infections in patients with COVID-19, including those with severe disease receiving intensive care [16] and with application of different cut-off values [17]. As in our cohort, the most useful performance parameter in the detection of bacterial infections was the high negative predictive value, mostly greater than 90–95% [16, 17], suggesting that procalcitonin is most useful in excluding bacterial infections and facilitating withholding or early cessation of antibiotics.

Procalcitonin has been evaluated as a component of antibiotic stewardship programmes to guide antibiotics prescription in patients hospitalized with COVID-19. When a procalcitonin-guided antibiotics prescription protocol was communicated repeatedly with physicians, and deviation from the protocol was requested for an explanation of decision, hospitals with access to procalcitonin testing had 47–67% lower chance of antibiotics initiation without compromising patients’ outcomes [18]. Procalcitonin testing within 72 h of antibiotics initiation for respiratory tract infections only was evaluated in a hospital in UK, where recommendations for continuation or discontinuation of antibiotics according to procalcitonin results were made, and procalcitonin testing was found to be associated with shorter duration of antibiotics and lower consumption [19]. Other studies have shown that low procalcitonin level enabled antibiotics to be withheld or stopped early in 73–99% of patients, when procalcitonin testing was included as part of a structured antibiotics stewardship programme, involving short turnaround time, well-defined testing indications, recommendations on procalcitonin-guided antibiotics prescription, and dedicated members of multidisciplinary antibiotic stewardship teams providing feedback to clinicians [20, 21].

On the other hand, in our cohort, procalcitonin testing was available, but there were no standardized protocols for the indication of the test, and the use of antibiotics according to procalcitonin results. In our cohort, procalcitonin testing was associated with higher antibiotics exposure and prescription at both patient and hospital level. Patients with procalcitonin testing were older, had more comorbidities, higher neutrophil and C reactive protein, and higher prevalence of documented bacterial infections. This suggests that procalcitonin testing was preferentially performed for patients with higher clinical suspicion of bacterial infections, rather than for the purpose of excluding bacterial infections [20].

On the other hand, patients in our cohort with a low level of procalcitonin had a lower probability of antibiotics initiation and shorter duration of antibiotics therapy. However, only 12% had antibiotics stopped within 2 days of performing the procalcitonin tests. Such findings were likely explained by variable turnaround time of procalcitonin testing among different hospitals, the lack of well-defined recommendations in procalcitonin-guided prescription of antibiotics, and the lack of dedicated manpower within antibiotic stewardship teams to provide feedback in response to procalcitonin results, especially during the COVID-19 waves. Our findings supported that procalcitonin testing should be incorporated into well-structured antibiotic stewardship programmes to optimize the effectiveness in promoting appropriate antibiotic initiation and cessation [22].

Based on our study findings and results of other studies evaluating the use of procalcitonin and antibiotics prescription in patients hospitalized with COVID-19, we propose that procalcitonin testing should be considered in patients when concomitant bacterial infections cannot be ruled out. The results of procalcitonin tests should be made available together with recommendations of antibiotics initiation, continuation or discontinuation according to procalcitonin results. Such practice should be clearly communicated with clinicians, and antibiotics stewardship strategies should be in place to facilitate appropriate adherence to those recommendations.

Our study had several limitations. The definition of bacterial infections was limited to documented bacterial infections with positive bacterial cultures. This may on one hand underestimate the prevalence of bacterial respiratory tract infections, which often failed to yield positive cultures despite extensive testing [1], and on the other hand, over-estimate urinary tract infections by including asymptomatic bacteriuria. Secondly, the prevalence of bacterial infections may have changed since 2020 due to increased use of corticosteroid and other immunosuppressive agents as standard of care for severe COVID-19 disease, although it was inconclusive whether the prevalence of bacterial infections would be significantly impacted by the use of these agents [6, 12]. Moreover, the most optimal cut-off value of procalcitonin in the detection of bacterial infections in patients with COVID-19 is uncertain. The cut-off of 0.5 ng/mL was chosen for this study, as the ability to exclude bacterial infections was shown to be similar for 0.25 ng/mL and 0.5 ng/mL [17, 21], and a higher cut-off value of 0.5 ng/mL has been recommended because of heightened inflammatory response in COVID-19 and a low prevalence of bacterial infections even with a higher cut-off [18].

## Conclusions

In conclusion, our study cohort demonstrated a low prevalence of bacterial infections among patients hospitalized with COVID-19 disease. Although antibiotics prescription was lower than in most published studies, unnecessary antibiotics prescription was observed. Procalcitonin testing was not associated with lower antibiotics prescription, although low procalcitonin level was associated with less exposure to antibiotics. The use of procalcitonin should be incorporated into a well-structured antibiotic stewardship programme to optimize antibiotics prescription in patients hospitalized for COVID-19.

### Electronic supplementary material

Below is the link to the electronic supplementary material.

Supplementary Material 1

## Data Availability

The datasets analysed during the current study are not publicly available but are available from the corresponding author on reasonable request.
