# Supplementary material for: Bacterial infections in patients with COVID-19: the impact of procalcitonin testing on antibiotics prescription in the real world
Source: BMC Infect Dis. 2024 Jan 19;24:106. doi: 10.1186/s12879-023-08849-x (PMC10797859; doi:10.1186/s12879-023-08849-x)
Supplement: Supplementary file 1 — Supplementary Material 1 [file 12879_2023_8849_MOESM1_ESM.docx]

**Supplementary Table 1. Causative bacteria of each type of infections**

|  |  | Pathogens | | | | | | | | |
| --- | --- | --- | --- | --- | --- | --- | --- | --- | --- | --- |
|  | Total number | *Staphylococcus* species | *Streptococcus* species | *Enterococcus* species | Enterobacterales | *Pseudomonas aeruginosa* | *Hemophilus influenzae* | *Acinetobacte*r species | *Stenotrophomonas maltophila* | Others |
| Bacterial co-infections | 147 |  |  |  |  |  |  |  |  |  |
| Respiratory | 68 | 21 | 5 | 0 | 19 | 7 | 5 | 2 | 0 | 9 |
| Urinary | 59 | 4 | 4 | 4 | 37 | 3 | 0 | 0 | 0 | 7 |
| Bloodstream | 12 | 7 | 1 | 0 | 2 | 0 | 0 | 0 | 0 | 2 |
| Others | 8 | 4 | 0 | 0 | 2 | 1 | 0 | 0 | 0 | 1 |
| Secondary bacterial infections | 222 |  |  |  |  |  |  |  |  |  |
| Respiratory | 120 | 24 | 4 | 0 | 39 | 21 | 10 | 7 | 5 | 10 |
| Urinary tract | 75 | 2 | 4 | 14 | 51 | 2 | 0 | 1 | 0 | 1 |
| Bloodstream | 14 | 6 | 0 | 0 | 6 | 0 | 0 | 0 | 0 | 2 |
| Others | 13 | 2 | 0 | 0 | 10 | 0 | 0 | 0 | 0 | 1 |

**Supplementary Table 2. Baseline characteristics and outcomes of patients with and without procalcitonin testing**

| Characteristics | Procalcitonin  N=2543 | No procalcitonin  N=6123 | P |
| --- | --- | --- | --- |
| Age (years) | 54.7±18.9 | 41.4±19.0 | <0.001 |
| Male | 1317 (51.8%) | 2884 (47.1%) | <0.001 |
| Any comorbidities | 1085 (42.7%) | 1246 (20.3%) | <0.001 |
| Hypertension | 618 (24.3%) | 598 (9.8%) | <0.001 |
| Diabetes | 389 (15.3%) | 319 (5.2%) | <0.001 |
| Obesity | 117 (4.6%) | 130 (2.1%) | <0.001 |
| Cardiovascular diseases | 185 (7.3%) | 171 (2.8%) | <0.001 |
| Neurological diseases | 128 (5.0%) | 142 (2.3%) | <0.001 |
| Liver diseases | 122 (4.8%) | 130 (2.1%) | <0.001 |
| Psychiatric disorders | 86 (3.4%) | 140 (2.3%) | 0.004 |
| Haematological disorders | 100 (3.9%) | 124 (2.0%) | <0.001 |
| Haematological and solid organ malignancy | 78 (3.1%) | 65 (1.1%) | <0.001 |
| Endocrine disorders | 55 (2.2%) | 67 (1.1%) | <0.001 |
| Pulmonary diseases | 45 (1.8%) | 72 (1.2%) | 0.035 |
| Rheumatological diseases | 62 (2.4%) | 55 (0.9%) | <0.001 |
| Renal diseases | 62 (2.4%) | 47 (0.8%) | <0.001 |
| Pregnancy | 11 (0.4%) | 23 (0.4%) | 0.699 |
| Immunocompromised conditions | 9 (0.4%) | 1 (0%) | <0.001 |
| Charlson comorbidity index | 2 (0, 3) | 0 (1, 0) | <0.001 |
| White cell count (x 10^9^/L) | 5.4 (4.3, 6.8) | 5.4 (4.3, 6.7) | 0.348 |
| Neutrophil count (x 10^9^/L) | 3.5 (2.6, 4.7) | 3.2 (2.4, 4.3) | <0.001 |
| C reactive protein (mg/L) | 0.93 (0.28, 3.50) | 0.31 (0.11, 0.88) | <0.001 |
| Lactate dehydrogenase (U/L) | 211 (178, 269) | 186 (161, 220) | <0.001 |
| Bilirubin (µmol/L) | 7.4 (5.3, 10.3) | 7.6 (5.6, 10.5) | 0.035 |
| Alanine transaminase (U/L) | 25 (17, 38) | 22 (15, 34) | <0.001 |
| Creatinine (µmol/L) | 72 (60, 89) | 68 (57, 81) | <0.001 |
| Intensive care | 349 (13.7%) | 60 (1.0%) | <0.001 |
| Bacterial infection | 263 (10.3%) | 63 (1.0%) | <0.001 |
| Bacterial co-infection | 64 (2.5%) | 32 (0.5%) |  |
| Secondary infection | 199 (7.8%) | 31 (0.5%) |  |
| Use of antibiotics during hospital stay | 1676 (65.9%) | 1097 (17.9%) | <0.001 |
| Duration of antibiotics therapy (days) | 11 (7, 19) | 7 (4, 10) | <0.001 |
| Length of antibiotics therapy | 9.5 (7, 15) | 7 (4, 8) | <0.001 |
| Length of stay in hospital (days) | 15 (11, 22) | 11 (7, 15) | <0.001 |
| Hospital death | 114 (4.5%) | 45 (0.7%) | <0.001 |
| Re-admission to hospital within 90 days | 220 (8.7%) | 380 (6.2%) | <0.001 |
